# Src Is a Potential Therapeutic Target in Endocrine-Resistant Breast Cancer Exhibiting Low Estrogen Receptor-Mediated Transactivation

**DOI:** 10.1371/journal.pone.0157397

**Published:** 2016-06-16

**Authors:** Stephanie K. Guest, Ricardo Ribas, Sunil Pancholi, Joanna Nikitorowicz-Buniak, Nikiana Simigdala, Mitch Dowsett, Stephen R. Johnston, Lesley-Ann Martin

**Affiliations:** 1 The Breast Cancer Now and Toby Robins Research Centre, Institute of Cancer Research, London, United Kingdom; 2 Royal Marsden Hospital, London, United Kingdom; II Università di Napoli, ITALY

## Abstract

Despite the effectiveness of endocrine therapies in estrogen receptor positive (ER+) breast cancer, approximately 40% of patients relapse. Previously, we identified the Focal-adhesion kinase canonical pathway as a major contributor of resistance to estrogen deprivation and cellular-sarcoma kinase (c-src) as a dominant gene in this pathway. Dasatinib, a pan-src inhibitor, has recently been used in clinical trials to treat ER+ patients but has shown mixed success. In the following study, using isogenic cell line models, we provide a potential explanation for these findings and suggest a sub-group that may benefit. A panel of isogenic cell lines modelling resistance to aromatase inhibitors (LTED) and tamoxifen (TAMR) were assessed for response to dasatinib ± endocrine therapy. Dasatinib caused a dose-dependent decrease in proliferation in MCF7-TAMR cells and resensitized them to tamoxifen and fulvestrant but not in HCC1428-TAMR. In contrast, in estrogen-deprived conditions, dasatinib increased the proliferation rate of parental-MCF7 cells and had no effect on MCF7-LTED or HCC1428-LTED. Treatment with dasatinib caused a decrease in src-phosphorylation and inhibition of downstream pathways, including AKT and ERK1/2 in all cell lines tested, but only the MCF7-TAMR showed a concomitant decrease in markers of cell cycle progression. Inhibition of src also caused a significant decrease in cell migration in both MCF7-LTED and MCF7-TAMR cells. Finally, we showed that, in MCF7-TAMR cells, in contrast to tamoxifen sensitive cell lines, ER is expressed throughout the cell rather than being restricted to the nucleus and that treatment with dasatinib resulted in nuclear shuttling of ER, which was associated with an increase in ER-mediated transcription. These data suggest that src has differential effects in endocrine-resistant cell lines, particularly in tamoxifen resistant models, with low ER genomic activity, providing further evidence of the importance of patient selection for clinical trials testing dasatinib utility in ER+ breast cancer.

## Introduction

Over 80% of breast cancers (BC) are estrogen receptor (ER) positive (+) at primary diagnosis. Estrogen (E) mediates its effects by binding to the ER. E-bound ER associates classically with E-response-elements (EREs) on target genes controlling proliferation and cell survival. ER has also been shown *in-vitro* to function via non-genomic mechanisms by association with growth factor signalling pathways [1].

The reliance of ER+ tumours on E has been exploited clinically by the development and use of various endocrine therapies, such as: aromatase inhibitors (AI), which block the conversion of androgens to estrogens; selective ER modulators (SERM), such as tamoxifen, which compete with E for the ER; and fulvestrant (ICI182780), which once bound, potentiates degradation of ER [2]. Despite the effectiveness of these current therapies, BC cells can circumvent the need for steroid hormones and resistance commonly occurs in approximately 40% of women, prioritizing the need to identify therapies to overcome this.

Using global gene expression data derived from cells adapted to long-term-E-deprivation (LTED), we showed that the Focal adhesion kinase (FAK) pathway, was one of the major pathways upregulated at the point of resistance and revealed cellular sarcoma kinase (c-src), as the major gene elevated in this pathway [3].

Src family kinases interact with a plethora of cellular cytosolic, nuclear and membrane proteins and modify these proteins by phosphorylation on tyrosine residues. Previous studies have alluded to the role of src in endocrine-resistant BC. For instance, the interaction between ER, src and the p85 subunit of PI3K leads to phosphorylation of AKT and ERK1/2, resulting in recruitment of PELP/MNAR to the ER nuclear transcription complex, promoting cell proliferation [4–6]. Previous studies have also shown that increased src activity after long-term treatment with tamoxifen enhances cellular invasion and motility in BC cells [7] and that impeding src activity reverses tamoxifen resistance [8].

Taken together, these data provide support for the role of src signalling in endocrine-resistant BC and provide a rationale for inhibiting src signalling in combination with endocrine therapy to circumvent or delay the development of endocrine-resistance.

Dasatinib is a potent orally available inhibitor of multiple oncogenic tyrosine kinases including the src family [9]. Recently, clinical trials using single agent dasatinib in solid tumors have shown limited activity [10–12]. The use of dasatinib in combination with endocrine therapy has also been investigated, however, dasatinib with fulvestrant or exemestane, did not show any benefit in progression free survival (PFS) for women with metastastic BC. In contrast, in a second study, the combination of dasatinib and letrozole did show improved PFS in women with ER+, HER2- metastastic BC (reviewed by [13]). In the following manuscript, we provide an explanation for these conflicting findings, highlighting the need for careful patient selection, in order to gain maximum clinical benefit from such combination therapies.

## Material and Methods

### Antibodies and reagents

Primary antibodies against phospho-src^tyr416^, phospho-src^tyr527^, src, phospho-ERK1/2 (p42/44), phospho-p90^rsk^, phospho-AKT^ser473^, total-AKT, cyclin D1, phospho-EGFR^tyr1068^, total-EGFR and total p27 were purchased from Cell Signalling; total-ER and phospho-p27^ser10^ from Santa Cruz; total ERK1/2 and Actin (Sigma Aldrich) and PgR (Novacastra). Total-ERBB2 and phospho-ERBB2^Tyr1248^ were purchased from Millipore. Secondary horseradish peroxidase antibodies were obtained from Dako. 17-β-estradiol (E2) and 4-hydroxytamoxifen (4-OHT) were purchased from Sigma-Aldrich, UK; fulvestrant (ICI182780) from Tocris Bioscience and dasatinib from Selleckchem.

### Tissue Culture

MCF7 and HCC1428 cells were obtained from the American Type Culture Collection (ATCC, Rockville, USA) and cultured in phenol red-free RPMI1640 medium supplemented with 10% fetal bovine serum and 1nM estradiol (E2). All cell lines were banked in multiple aliquots to reduce the risk of phenotypic drift and identity confirmed by short tandem repeat profiling (Promega, Madison, WI, USA). Long-term-estrogen-deprived cells (MCF7-LTED and HCC1428-LTED) modelling resistance to an AI were cultured in phenol red free RPMI 1640 supplemented with 10% DCC-FBS [14]. MCF7-TAMR and matched MCF7 cells were maintained in DMEM-F12 lacking phenol red and supplemented with 1% FBS and containing 100nM 4OHT, as previously described [15, 16]. The parental cell line was referred to as 1%MCF7 and is a recognised model which is refractory to E2 but sensitive to tamoxifen and fulvestrant [15, 17], HCC1428-TAMR were cultured in the presence of 0.01nM E2 and 100nM 4-OHT. Cells were stripped of E2 or 4-OHT for 3 days prior to experimentation.

### Proliferation assays

Cells lines were seeded in 96-well plates. Monolayers were allowed to acclimatise for 24-hours prior to treatment with drug concentrations and combinations, as indicated, for 6-days with a medium change on day 3. Cell viability was determined using CellTitre-Glo^®^ Luminescent Cell Viability Assay (Promega), according to the manufacture’s instructions.

### Transcriptional assays

Cell lines were seeded in 24-well plates and allowed to acclimatise for 24 hours. Subsequently, monolayers were transfected using *Trans*IT-LT1 transfection reagent (Mirus) with 0.1μg of ER reporter construct (EREtkluc) and 0.1μg of pCH110 overnight. The following day, cells were treated with the drug combinations specified and left for 24-hours. Luciferase (Promega) and β-galactosidase (GalactoStar, Applied Biosystems) activities were measured using a luminometer (VICTOR^™^ X5 Multilabel Plate Reader). Each experiment was performed 3 times with 3–4 replicates per treatment.

### Western Blotting

Cells were seeded into dishes and allowed to attach overnight. Monolayers were treated with the desired drug combinations for the required length of time. Whole-cell extracts were generated, as described previously [14]. Densitometric evaluation was performed using ImageJ.

### Real-time quantitative PCR

mRNA was extracted from treated cells using the RNeasy Mini kit (Qiagen) according to the manufacturers instructions and quantified using the Agilent2100 Bioanalyzer (Expert Software version B.02.03) with RNA Nano LabChip Kits (Agilent Technologies, Wokingham, Berkshire, UK). Total RNA was reverse transcribed using SuperScript III (Invitrogen) and random primers, in accordance with the manufacturer's instructions. qRT-PCR was performed in triplicate using the ABI Perkin-Elmer Prism 7900HT Sequence detection system (Applied Biosystems). Taqman gene expression assays (Applied Biosystems) were used to detect expression of uPA (Hs01547051_m1) and Caveolin-1 (Hs00971716_m1), together with FKBP15 (Hs00391480_m1), as a housekeeping gene, to normalise the data. The relative quantity was determined using ΔΔct according to the manufacturer's instructions (Applied Biosystems).

### Cell migration assay

Boyden chambers (8μm pores) were placed into 24-well plates containing normal growth media and 10ng/ml EGF, as a chemo attractant. MCF7-LTED and MCF7-TAMR cells were pre-treated with 100nM dasatinib for 1-hour and then seeded at a density of 5x10^4^ in the upper chamber in serum free media for 48-hours. Non-migrating cells were scraped from the top surface of the membrane while migrated cells were stained with Harris’ Haematoxylin (VWR). The membranes were excised and mounted on a microscope slide and the number of migrating cells counted.

### Immunofluorescence and confocal microscopy

Cells were seeded on glass coverslips in basal media and treated for 24-hours with vehicle or 100nM dasatinib, as indicated. Monolayers were fixed with 4% paraformaldehyde in PBS for 15 minutes and permeabilized with 0.5% triton X100 for 10 minutes. Cells were subsequently blocked with PBS containing 1%BSA and 2%FBS and incubated with primary antibodies against psrc^tyr416^ (Cell Signalling) and ER (Santa Cruz) for 2-hours. Cells were incubated with Alexa fluor 488 or 546 (red)-labelled secondary antibodies (Molecular Probes) for 1-hour and nuclei counterstained with DAPI (Invitrogen). Coverslips were finally mounted onto glass slides using vectashield-mounting media (Vector Labs) and images collected on the Zeiss LSM710 confocal microscope.

### Statistical Analysis

Statistical analysis was conducted using one-way and two-way ANOVA to assess the significance of either escalating concentrations of dasatinib or the combination effects with other endocrine agents. Student’s t-test was used for the qRT-PCR, migration and transcription assays.

## Results

### Differential sensitivity of isogenic cell lines to dasatinib

**Fig 1:**
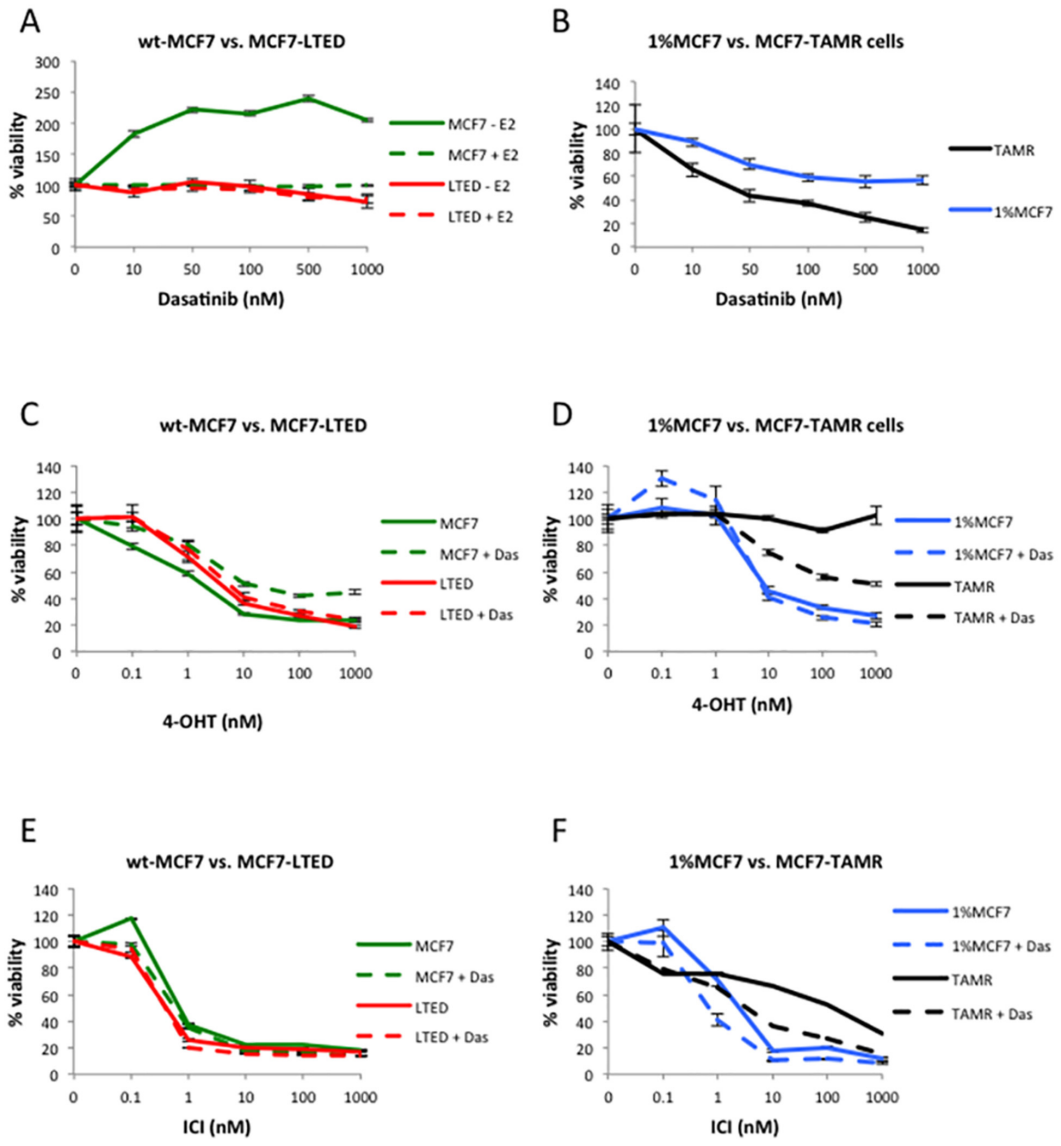
Antiproliferative effects of dasatinib alone or in combination with endocrine agents. Cells were treated with increasing concentrations of dasatinib (**A,B**), or fixed concentration of dasatinib and increasing concentrations of 4-OHT (**C,D**) or fulvestrant (ICI) (**E,F**) for 6-days with a medium change at day 3. Cell viability was determined using CellTitre-Glo^®^ Luminescent Cell Viability Assay. The data are representative of three independent experiments (Error bars represent ± SEM).

Src is regulated by phosphorylation of two tyrosine residues, which have opposing effects. Tyrosine 416 in the activation loop of the src kinase domain increases enzyme activity, whilst phosphorylation of tyrosine 527 by Csk reduces activity of the enzyme. Dasatinib is know to target both phosphorylated sites [18]. Assessment of the expression of src in a panel of isogenic cell lines modelling resistance to E-deprivation (MCF7-LTED, HCC1428-LTED) or tamoxifen (MCF7-TAMR, HCC1428-TAMR) showed a marked increase in total src expression in MCF7 models of endocrine resistance, in contrast to a decrease seen in the HCC1428 derivatives. Concordant expression levels of phosphorylated src were also evident (S1A Fig). Based upon this finding, all cell lines were treated with escalating doses of dasatinib in the presence or absence of 0.01nM E2 (Fig 1A and 1B). Increasing concentrations of dasatinib showed no effect on proliferation in wt-MCF7 cells in the presence of E2, however, strikingly, in the absence of E2, dasatinib caused a 2-fold dose-dependent increase in proliferation (p<0.0001). MCF7-LTED showed no significant response to the antiproliferative effect of dasatinib in the presence or absence of E2 compared to vehicle control (Fig 1A). In contrast, the 1%MCF7 cell line, showed a slight but noticeable concentration dependent decrease in proliferation (p<0.0001), although this did not reach an IC_50_ value even at concentrations as high as 1000nM. The MCF7-TAMR cell line showed the highest degree of sensitivity with an IC_50_ of c.25nM (p<0.0001) (Fig 1B). Of note, the wt-HCC1428, as well as the LTED and TAMR derivatives, which showed low src expression, were refractory to the inhibitory effect of dasatinib (S1A and S1B Fig) suggesting this pathway may not play a significant role in these cell lines.

We further assessed the effect of dasatinib in combination with increasing concentrations of 4-OHT (Fig 1C and 1D; S1C Fig) and fulvestrant (Fig 1E and 1F). 4-OHT and fulvestrant caused a dose-dependent decrease in proliferation of wt-MCF7 and MCF7-LTED. Addition of dasatinib reduced the sensitivity of wt-MCF7 cells to the antiproliferative effect of 4-OHT by 10-fold (1nM to 10nM, p<0.0001), but showed no significant added or deleterious effect in the MCF7-LTED (Fig 1C). No additional benefit of the combination of fulvestrant with dasatinib was observed in MCF7-LTED, however in wt-MCF7 although no marked benefit was evident, a statistical difference was detected (p<0.0001) (Fig 1E). As expected, the proliferation of the MCF7-TAMR cell line was unaffected by escalating concentrations of 4-OHT, however, the addition of dasatinib re-sensitised to the SERM (IC_50_ 1000nM, p<0.0001). 1%MCF7 cells showed no significant additional benefit of the combination of 4-OHT with dasatinib (Fig 1D). Finally, we evaluated the effect of fulvestrant in combination with dasatinib in 1%MCF7 and TAMR cell lines. Both cells lines showed increased sensitivity to fulvestrant when combined with dasatinib (IC_50_ 8nM to 1nM in 1%MCF7, p = 0.0003 vs. 100nM to 5nM in TAMR, p<0.0001) (Fig 1F). In contrast to these observations, HCC1428-TAMR showed no response to 4-OHT in the presence or absence of dasatinib (S1C Fig) and were therefore excluded from further analysis.

### Effect of dasatinib and endocrine therapy on cell signalling pathways and cell cycle

**Fig 2:**
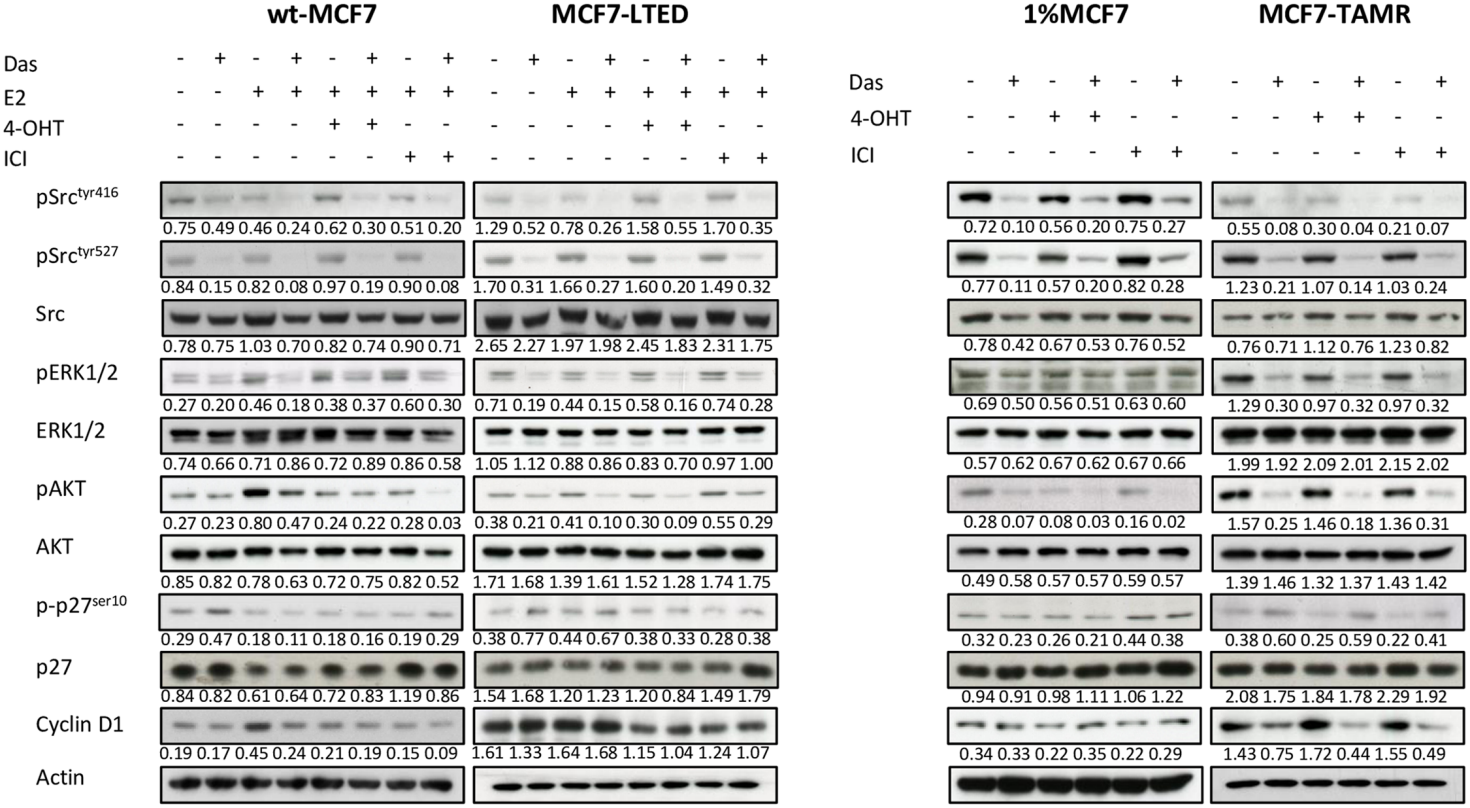
Analysis of src and downstream signalling in response to dasatinib combined with endocrine agents. Wt-MCF7, MCF7-LTED, 1%MCF7 and MCF7-TAMR cells were treated for 24-hours with the drug combinations mentioned above. Standard concentrations of dasatinib (100nM), E2 (0.01nM), 4-OHT (10nM) and fulvestrant (ICI) (10nM) were used. Whole-cell extracts were assessed for expression of various proteins indicated using immunoblotting. Figures below each panel represent semi-quantitave changes in protein expression relative to actin.

In order to ascertain the effect of dasatinib in combination with endocrine agents on cellular signal transduction pathways, cells were treated with dasatinib alone and in combination with 4-OHT or fulvestrant for 24-hours (Fig 2). Dasatinib alone caused a dose-dependent decrease in phosphorylation of src at tyrosine 416 and 527 in all cell lines tested (S1D Fig). Of note, low concentrations of dasatinib (10nM) caused a slight but noticeable increase in psrc^tyr416^ but not psrc^tyr527^, suggesting the activation of a negative feedback loop, which is negated at higher concentrations.

Based on this observation, together with our proliferation assays and previous publications [19, 20] we selected a concentration of 100nM for further *in vitro* studies. The combination of dasatinib with endocrine agents (4-OHT and fulvestrant) showed inhibition of src phosphorylation in all endocrine-sensitive and resistant cell lines. Expression of phosphorylated ERK1/2 and phosphorylated AKT was also inhibited by dasatinib ± the endocrine agents, but was most pronounced in the MCF7-TAMR cells, whilst total-ERK1/2 and AKT remained unchanged.

We further assessed the effect of dasatinib on cell cycle markers. Dasatinib increased phosphorylation of p27 in wt-MCF7 in the absence of E2 and in combination with fulvestrant. Similarly, dasatinib caused a slight but noticeable increase in MCF7-LTED although this did not translate into a marked antiproliferative effect as seen in our combination studies. MCF7-TAMR cells following treatment with dasatinib showed significant increases in p27 phosphorylation together with a substantial down-regulation of cyclinD1, contrasting to the other cell lines tested (Fig 2 and S2 Fig). These data are in keeping with the proliferation assays, which showed that dasatinib was most effective in the MCF7-TAMR cell line.

### Dasatinib increases ER mediated transcription in isogenic cell lines

**Fig 3:**
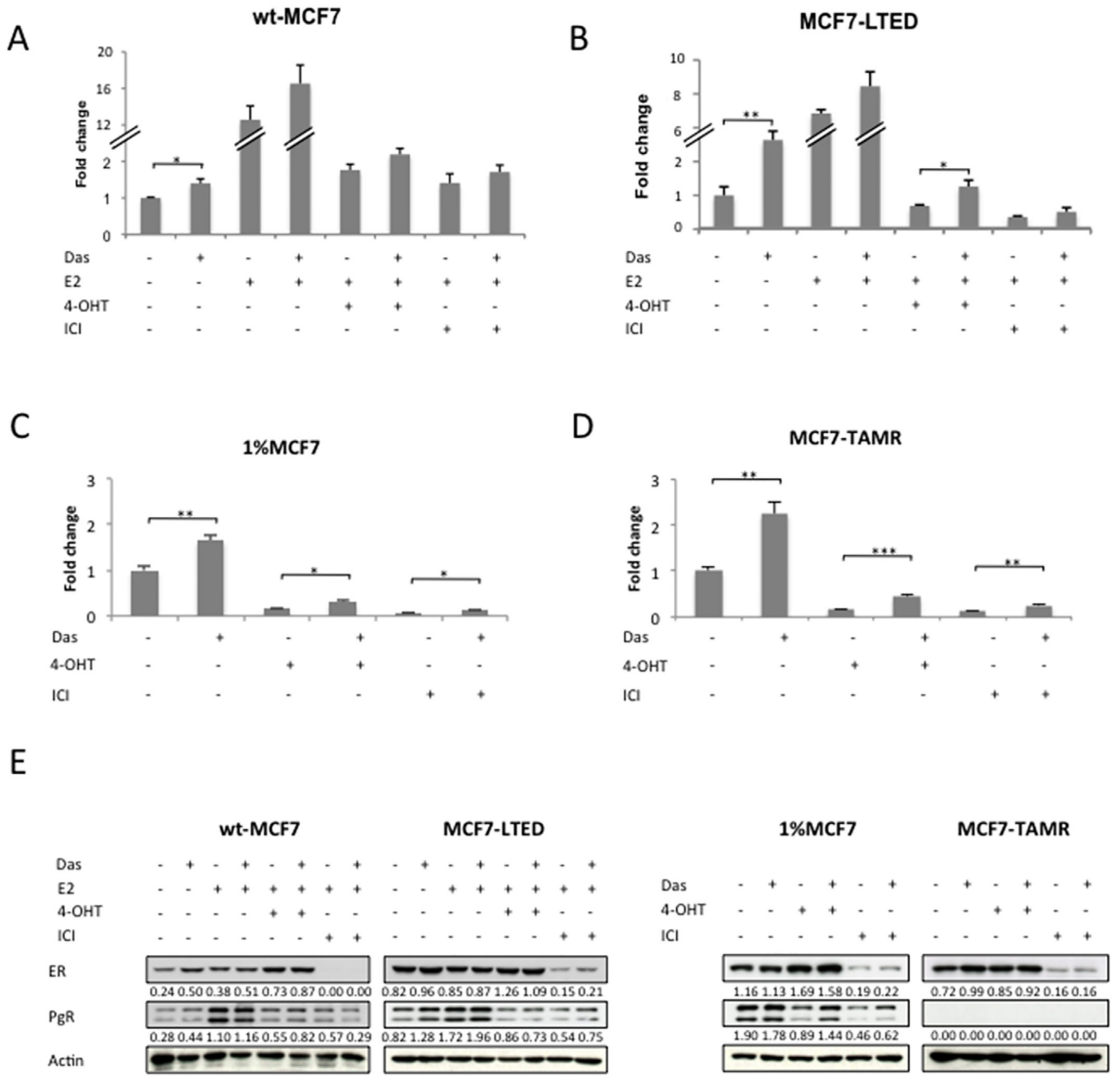
Effects of dasatinib on ER-mediated transcription and ER signalling. Cell lines were co-transfected with EREIItkLuc and pCH110 and treated with the combinations mentioned (**A, B, C, D**). Standard concentrations of dasatinib (100nM), E2 (0.01nM), 4-OHT (10nM) and fulvestrant (ICI) (10nM), were used. Normalized luciferase activity from triplicate wells was expressed relative to the vehicle-treated control. Bars represent ± SEM. *p<0.05, **p<0.01, ***p<0.001. Effects were confirmed in three independent experiments. Wt-MCF7, MCF7-LTED, 1%MCF7 and MCF7-TAMR cells were treated as indicated for 24-hours, with the same amounts as stated above. Western blot was used to assess changes in total ER and PgR. Figures below each panel represent semi-quantitave changes in protein expression relative to actin.(**E**).

We further analysed the effect of dasatinib on ER-mediated transactivation using an ERE-luciferase-reporter construct. Dasatinib alone or in combination with the endocrine agents caused a significant increase in ER-mediated transactivation in both the MCF7-TAMR and 1%MCF7 cells. This increase was also noted in the MCF7-LTED although this only met significance in the absence of exogenous E2 or in the presence of 4-OHT (Fig 3A–3D). In keeping with the ER-mediated transactivation data, PgR expression was increased by the addition of dasatinib in the absence of E2 in wt-MCF7 and MCF7-LTED. Similarly, dasatinib induced expression of PgR in the 1%MCF7 in the presence of both 4-OHT and fulvestrant. The MCF7-TAMR cells, in keeping with previous data [21], did not express PgR and this did not change with addition of dasatinib (Fig 3E and S2 Fig).

### Expression of uPA and caveolin-1 are not biomarkers of sensitivity

**Fig 4:**
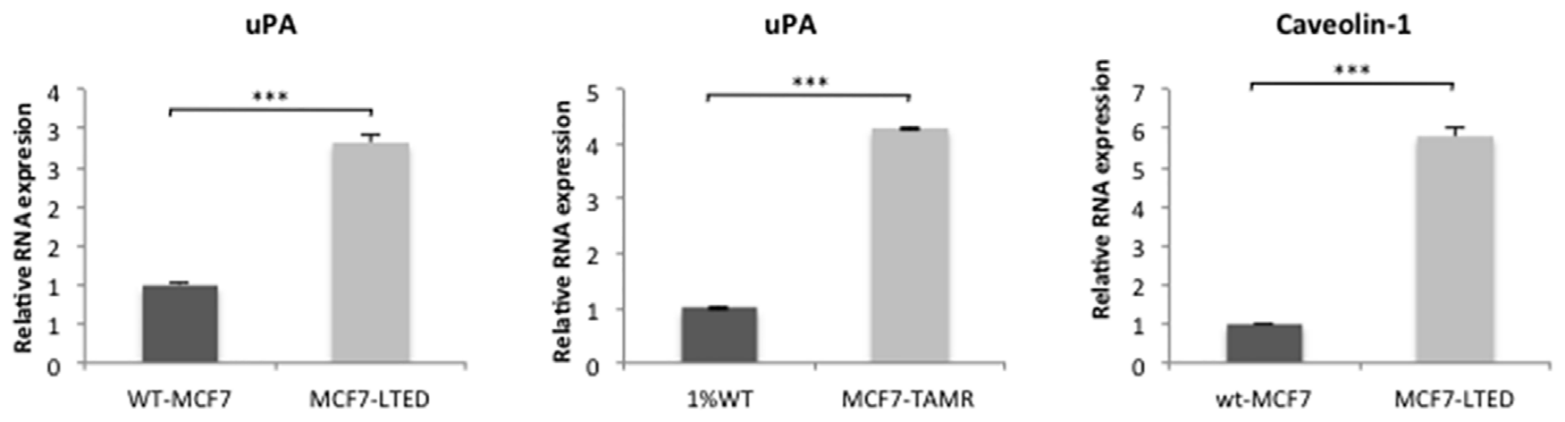
Expression of uPA and Caveolin-1 in endocrine-resistant versus sensitive cell lines. Assessment of mRNA relative expression of uPA and Caveolin-1 using qPCR in wt-MCF7, MCF7-LTED, 1%MCF7 and MCF7-TAMR cell lines. Bars represent ± SEM. *p<0.05, **p<0.01, ***p<0.001.

It has previously been reported, that sensitivity to dasatinib is dependent on BC subtype, with basal and post-epithelial-mesenchymal transition (EMT) BC cell lines, showing highest sensitivity [22, 23]. Dasatinib sensitivity has also been associated with expression of caveolin-1 and urokinase plasminogen activator (uPA) [22–26]. To test the expression of these potential biomarkers, qRT-PCR was performed. MCF7-LTED and MCF7-TAMR cells both showed increased levels of uPA compared to their respective parental cell line. Expression of caveolin-1 was also higher in the MCF7-LTED (Fig 4A). In contrast, 1%MCF7 and MCF7-TAMR showed no expression (data not shown). In accordance with previous data [23, 26], treatment with dasatinib reduced mRNA expression of both uPA and caveolin-1 (S3 Fig), suggesting neither were the defining feature of sensitivity in these models.

### Dasatinib inhibits migration of endocrine-resistant cell lines

**Fig 5:**
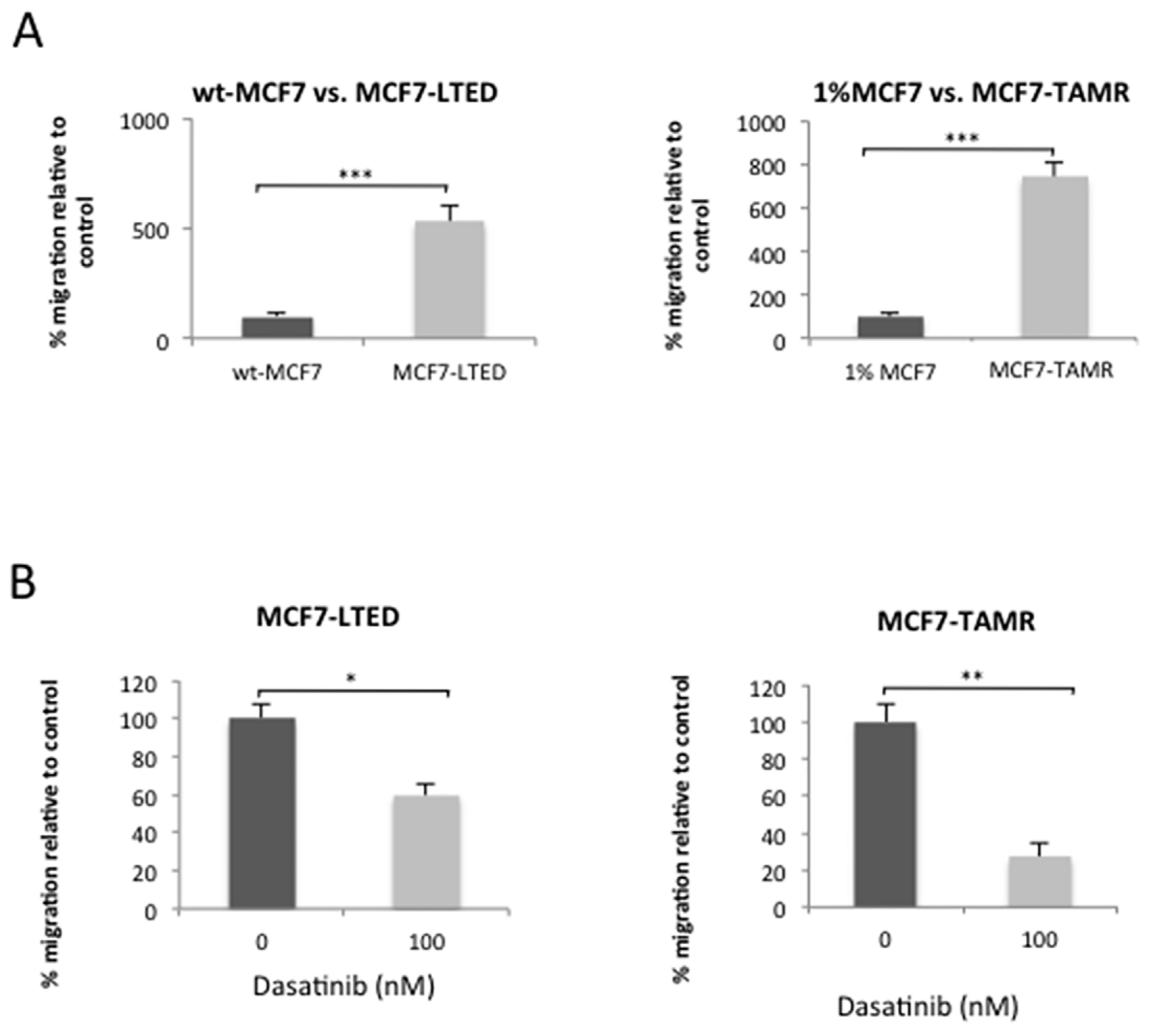
Effect of dasatinib on basal migration. Migratory capacity of endocrine-resistant cell lines (MCF7-LTED and MCF7-TAMR) compared to endocrine-sensitive cells lines (wt-MCF7 and 1%MCF7, respectively) (**A**); Effect of dasatinib upon migratory capacity in MCF7-LTED and MCF7-TAMR cell lines (**B**). Bars represent ± SEM. *p<0.05, **p<0.01, ***p<0.001.

We further assessed the effect of dasatinib on the anti-migratory potential. Both MCF7-LTED and TAMR cells showed higher migratory capability when compared to their parental cell lines (5.3-fold versus 7.5-fold increase, respectively) (Fig 5A). Dasatinib caused a 40% reduction in the migration of MCF7-LTED and 73% in the MCF7-TAMR cells (Fig 5B).

### MCF7-LTED and MCF7-TAMR cells have similar baseline features but differ in the level of ER-mediated transactivation

Having established that dasatinib had differential effects in the endocrine-resistant cell lines, we sought to investigate the similarities and differences between these isogenic models. Of note, the features of MCF7-LTED and MCF7-TAMR were concordant, both expressing higher levels of ERα, ERBB2, pERK1/2 and p90rsk compared to their parental control (S4A Fig), but differed with regard to ER-mediated transactivation [14, 21]. The MCF7-LTED showed a 7.8-fold increase in basal transcription compared to wt-MCF7 in the absence of E2, the MCF7-TAMR cells showed 5.6-fold lower level compared with the 1%MCF7 (S4B Fig). Moreover, while MCF7-TAMR cells continue to express ERα similar to MCF7-LTED, they lose the expression of PgR in support of their lower ER genomic activity (S4A Fig).

### Dasatinib treatment results in nuclear shuttling of ER in MCF7-TAMR cells

**Fig 6:**
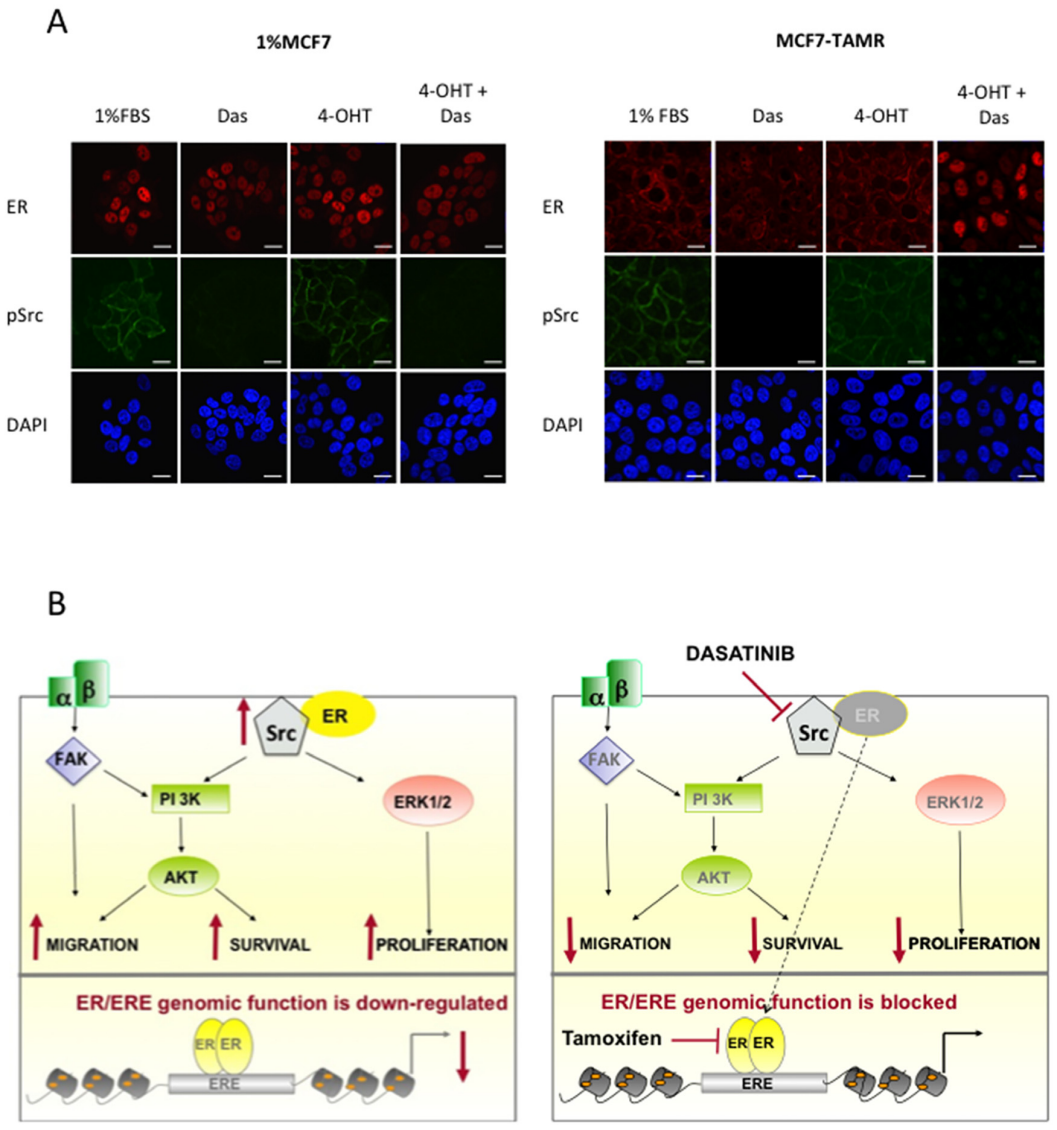
Dasatinib promotes nuclear shuttling of ER in the MCF7-TAMR cells but not the 1%MCF7. 1%MCF7 and MCF7-TAMR cells were treated for 24-hours with 1%FBS, dasatinib (100nM), 4-OHT (10nM), or the combination and stained with ER, pSrc^tyr416^ and DAPI; bars indicate 20μm **(A)**. Schematic diagram showing cross-talk between non-genomic ER and Src. ER associates with Src at the cell membrane via a non-genomic mechanism. This leads to an increase in both ERK1/2 and AKT providing a survival advantage. The reduced genomic activity of ER in this setting enhances resistance to tamoxifen. Inhibition of Src with dasatinib causes ER to shuttle to the nucleus where it is targeted by tamoxifen, leading to a decrease in proliferation and re-sensitization to the endocrine agent **(B)**.

We have previously shown that ER acts via a non-genomic mechanism in the MCF7-TAMR cells and that they appear to have an impeded luminal phenotype in relation to ER genomic function. Contrastingly the wt-MCF7, MCF7-LTED and 1%MCF7 retain classical nuclear ER function [21] (S5 Fig). We, therefore hypothesised that src may be involved in cellular localisation of ER in the MCF7-TAMR phenotype. To test this, we treated 1%MCF7 and MCF7-TAMR cells with dasatinib alone and in combination with 4-OHT. In the presence of 4-OHT alone, there was no change in the localisation of ER in MCF7-TAMR cells, whereas the combination of tamoxifen and dasatinib resulted in ER being shuttled into the nucleus (Fig 6A).

## Discussion

Acquisition of endocrine-resistance is a common occurrence in BC patients and the identification of appropriate clinical interventions remains of paramount importance. Despite multiple drugged targets being proposed from *in-vitro* studies; data from resulting clinical trials has failed to meet expectation. For example, src activation has been associated with adaptation to both tamoxifen and LTED. Studies suggest that interaction between Cas and c-Src through pathways involving EGFR and signal transducer and activator of transcription 5b (STAT5b) are pivotal to this resistance mechanism [7, 27]. Furthermore, Hiscox and colleagues have provided evidence that perturbation of src signalling reduces the invasive phenotype of tamoxifen resistant MCF7 cells [7]. To date, three predictive gene signatures derived from *in vitro* studies have been reported [24, 28, 29] but have failed to identify a group of BC patients for whom single agent dasatinib is effective [30]. Of note, gene signatures do not necessarily take into consideration cellular context such as protein trafficking [31],

Clinical trials assessing the combination of dasatinib with letrozole, exemestane or fulvestrant have provided mixed results. For instance, addition of dasatinib to fulvestrant or exemestane did not provide any benefit in PFS in women with metastatic BC [32, 33], in contrast Paul et al showed that the combination of letrozole and dasatinib increased PFS from 9.9 months in the letrozole alone arm to 20.1 months in the combination [34]. One explanation for these contrasting results, is the difference in the patient cohorts. In the trials investigating fulvestrant and exemestane, the majority of patients had relapsed on an AI, whilst those in Paul’s study had relapsed on tamoxifen. It is tempting to hypothesise, that the mechanism of resistance to the endocrine agent subsequently governs sensitivity to dasatinib. To investigate this further, we compared the effect of dasatinib in combination with endocrine therapy in a panel of isogenic cell lines modelling acquired resistance to either tamoxifen or LTED.

In this study, we showed that dasatinib had different effects on endocrine-resistant and sensitive cell lines. Of note, in the absence of exogenous E (modelling patients receiving an AI) the addition of dasatinib promoted, rather than impeded the proliferation of wt-MCF7 cells and the same effect was seen with 4-OHT, suggesting the combination would be deleterious in the primary setting. A potential mechanism related to this observation is discussed below. Furthermore, no benefit was seen in MCF7-LTED suggesting the combination of dasatinib with E-deprivation, 4-OHT or fulvestrant would provide no further benefit in the metastatic setting. Similarly, HCC1428-LTED and HCC1428-TAMR were resistant to the antiproliferative effect of dasatinib, possibly as a result of the reduction in expression of src compared to wt-HCC1428. In contrast, dasatinib reduced the proliferation of the MCF7-TAMR cell line, increased sensitivity to fulvestrant and re-sensitised them to the antiproliferative effect of 4-OHT. These data were further supported by downstream analysis of cell cycle proteins, which showed that dasatinib increased phosphorylated p27 and decreased cyclinD1 most significantly in the MCF7-TAMR cell line. In contrast, both the wt-MCF7 and MCF7-LTED showed little difference when comparing single agent endocrine therapy with or without the addition of dasatinib,

Previous studies have associated over-expression of uPA and CAV1 with tamoxifen resistance [35–37] and as determinant of sensitivity to dasatinib [22, 25]. However, no differential expression of uPA was evident between the two resistant cell lines and whilst the LTED showed a marked increase in expression of CAV1, the MCF7-TAMR showed no expression in keeping with previous studies [26, 38]. Furthermore, both resistant cell lines showed an enhanced migratory phenotype compared to their parental line and in both cases dasatinib impeded migration to a similar degree.

To address the discordance in the antiproliferative effect of dasatinib in the LTED versus the MCF7-TAMR cell line, we concentrated on differences in phenotype. Analysis showed that expression of key receptor tyrosine kinases and their downstream partners was consistent between the two resistant models. However, the MCF7-TAMR cell line showed over a 5-fold reduction in ER genomic activity and loss of PgR expression, whereas in the LTED model ER genomic activity was 5-fold higher than their parental control, although PgR expression was significantly reduced. This suggests that the ER in the LTED setting remains classical in its function as a genomic transcription factor.

Previous studies have provided support for interplay between ER and src, resulting in phosphorylation of the ER at tyrosine 537 and promoting nuclear export. Inhibition of src negates this effect, leading to sequestration of ER in the nucleus [39, 40]. This could account for the growth promoting effect of dasatinib in the wt-MCF7 in the absence of E2, allowing the retained nuclear receptor to continue to bind EREs promoting cell survival. This increase in transcription was also observed in the MCF7-TAMR cell line although it appeared insufficient to overcome upstream inhibitory effects on ERK1/2 and AKT leading to an overall drop in proliferation. One explanation for this is due to the cellular context. For instance, src and ER have been shown to interact at the cell membrane in complex with PELP-1 and PI3K, leading to activation of the ERK1/2 and AKT pathways [41, 42]. To address this, we performed confocal microscopy of ER in the LTED and MCF7-TAMR setting and showed that whilst ER in the LTED cells remained nuclear, in the MCF7-TAMR cell line it was both membranous and cytoplasmic. Treatment of the MCF7-TAMR cells with the combination of dasatinib and 4-OHT resulted in nuclear shuttling of ER, potentially allowing it to become a classical target for 4-OHT, which associated with a decrease in pAKT and pERK1/2, together with a significant reduction in proliferation.

Taken together, these data suggest that the mode of resistance influences sensitivity to dasatinib, and those tumours with an impeded luminal phenotype, in relation to ER-genomic function are most likely to respond (Fig 6B). This provides further evidence of the importance of patient selection for clinical trials testing dasatinib utility in ER-positive BC.

## Supporting Information

S1 Fig Src as a target of dasatinib. Expression of src in isogenic MCF7 and HCC1428 cell lines modelling resistance to LTED or tamoxifen **(A)**. Effect of increasing concentrations of dasatinib on proliferation of wt-HCC1428, HCC1428-LTED and HCC1428-TAMR in the presence or absence of exogenous E2 (**B**) assessment of the addition of dasatinib to re-sensitise HCC1428-TAMR to the antiproliferative effect of 4-OHT (**C**). Effect of increasing amounts of dasatinib upon phosphorylation and total src in wt-MCF7, MCF7-LTED, 1%MCF7 and MCF7-TAMR cell lines (**D**). Figures below each panel in the western blots represent semi-quantitave changes in protein expression relative to actin.
(PPTX)

S2 Fig Densitometric analysis of three independent studies showing mean arbitrary changes relative to DCC or 1% controls. (PPTX)

S3 Fig Effect of dasatinib upon mRNA expression of uPA in wt-MCF7, MCF7-LTED (A); 1%MCF7 and MCF7-TAMR cells (B); and Caveolin-1 in wt-MCF7 and MCF7-LTED cells (C). Bars represent ± SEM. *p<0.05, **p<0.01, ***p<0.001.
(PPTX)

S4 Fig Baseline expression of proteins in MCF7-LTED and MCF7-TAMR compared to their wt counterparts Figures below each panel represent semi-quantitave changes in protein expression relative to actin (**A**). ER/ERE transactivation in wt-MCF7, MCF7-LTED, 1%MCF7 and MCF7-TAMR cells (**B**). Bars represent ± SEM. *p<0.05, **p<0.01, ***p<0.001.
(PPTX)

S5 Fig ER localisation in wt-MCF7 versus MCF7-LTED derivatives under basal conditions, bars indicate 20 μm. (PPTX)
