# Supplementary material for: Src Is a Potential Therapeutic Target in Endocrine-Resistant Breast Cancer Exhibiting Low Estrogen Receptor-Mediated Transactivation
Source: PLoS One. 2016 Jun 16;11(6):e0157397. doi: 10.1371/journal.pone.0157397 (PMC4911087; doi:10.1371/journal.pone.0157397)
Supplement: S1 Fig — Expression of src in isogenic MCF7 and HCC1428 cell lines modelling resistance to LTED or tamoxifen (A). Effect of increasing concentrations of dasatinib on proliferation of wt-HCC1428, HCC1428-LTED and HCC1428-TAMR in the presence or absence of exogenous E2 (B) assessment of the addition of dasatinib to re-sensitise HCC1428-TAMR to the antiproliferative effect of 4-OHT (C). Effect of increasing amounts of dasatinib upon phosphorylation and total src in wt-MCF7, MCF7-LTED, 1%MCF7 and MCF7-TAMR cell lines (D). Figures below each panel in the western blots represent semi-quantitave changes in protein expression relative to actin. (PPTX) [file pone.0157397.s001.pptx]

## Slide 1
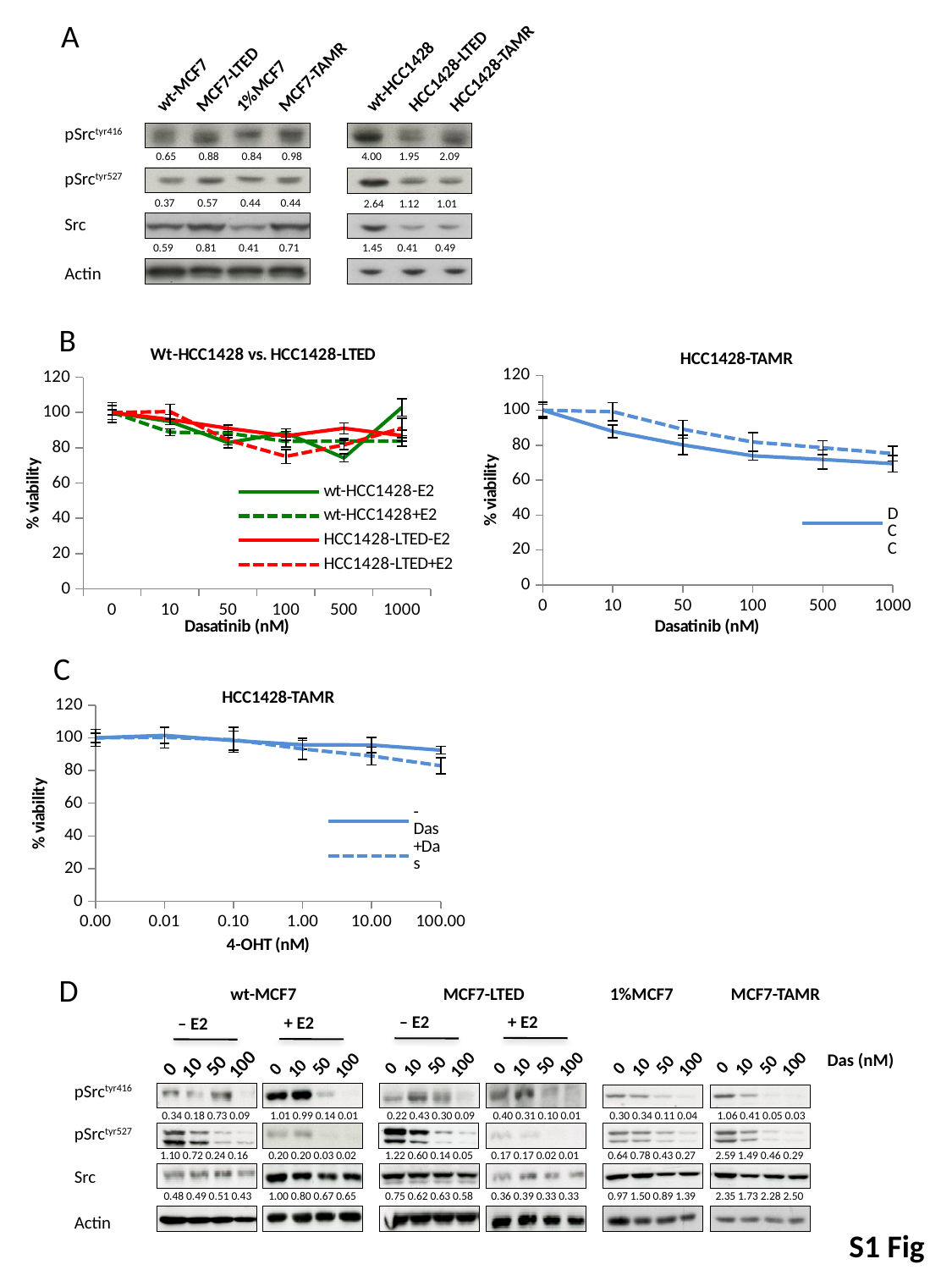

A
MCF7-LTED
1%MCF7
MCF7-TAMR
HCC1428-LTED
HCC1428-TAMR
wt-MCF7
wt-HCC1428
pSrctyr416
0.65 0.88 0.84 0.98
4.00 1.95 2.09
pSrctyr527
0.37 0.57 0.44 0.44
2.64 1.12 1.01
Src
1.45 0.41 0.49
0.59 0.81 0.41 0.71
Actin
B
### Chart
| Category | wt-HCC1428-E2 | wt-HCC1428+E2 | HCC1428-LTED-E2 | HCC1428-LTED+E2 |
|---|---|---|---|---|
| 0.0 | 100.0 | 100.0 | 100.0 | 100.0 |
| 10.0 | 94.87064083538073 | 88.8229890586515 | 96.03515474123132 | 100.490008807103 |
| 50.0 | 82.89853192012737 | 88.30564900441175 | 91.0416392296748 | 84.42255688500363 |
| 100.0 | 88.4455275058861 | 83.63298162428875 | 86.78205687088602 | 75.11977339099127 |
| 500.0 | 74.24359151103288 | 83.76364721032847 | 91.06382703584181 | 81.64965707944478 |
| 1000.0 | 102.9134532990574 | 83.86170534927192 | 86.94584507806736 | 91.2205061034398 |
### Chart
| Category | | |
|---|---|---|
| 0.0 | 100.0 | 100.0 |
| 10.0 | 87.87181060423099 | 99.16647090562725 |
| 50.0 | 80.10346838290808 | 89.07703869828671 |
| 100.0 | 73.90015208579467 | 81.78706582342598 |
| 500.0 | 71.7755200616176 | 78.50846605320051 |
| 1000.0 | 69.4163952403021 | 75.1842600783289 |HCC1428-TAMR
C
### Chart
| Category | | |
|---|---|---|
| 0.0 | 100.0 | 100.0 |
| 0.01 | 101.5021868470632 | 100.3200470443135 |
| 0.1 | 98.33393060349404 | 98.83924401245284 |
| 1.0 | 95.7582292301921 | 93.20888794060109 |
| 10.0 | 95.61079780623685 | 88.8810973190203 |
| 100.0 | 92.5003824624711 | 82.9465664546464 |HCC1428-TAMR
D
wt-MCF7
MCF7-LTED
1%MCF7
MCF7-TAMR
+ E2
100
50
0
10
– E2
100
50
0
10
+ E2
100
50
0
10
– E2
100
50
0
10
100
50
0
10
100
50
0
10
Das (nM)
pSrctyr416
0.34 0.18 0.73 0.09
1.01 0.99 0.14 0.01
0.22 0.43 0.30 0.09
0.40 0.31 0.10 0.01
0.30 0.34 0.11 0.04
1.06 0.41 0.05 0.03
pSrctyr527
1.10 0.72 0.24 0.16
0.20 0.20 0.03 0.02
1.22 0.60 0.14 0.05
0.17 0.17 0.02 0.01
0.64 0.78 0.43 0.27
2.59 1.49 0.46 0.29
Src
0.48 0.49 0.51 0.43
1.00 0.80 0.67 0.65
0.75 0.62 0.63 0.58
0.36 0.39 0.33 0.33
0.97 1.50 0.89 1.39
2.35 1.73 2.28 2.50
Actin
S1 Fig
