# Supplementary material for: Src Is a Potential Therapeutic Target in Endocrine-Resistant Breast Cancer Exhibiting Low Estrogen Receptor-Mediated Transactivation
Source: PLoS One. 2016 Jun 16;11(6):e0157397. doi: 10.1371/journal.pone.0157397 (PMC4911087; doi:10.1371/journal.pone.0157397)
Supplement: S2 Fig — (PPTX) [file pone.0157397.s002.pptx]

## Slide 1
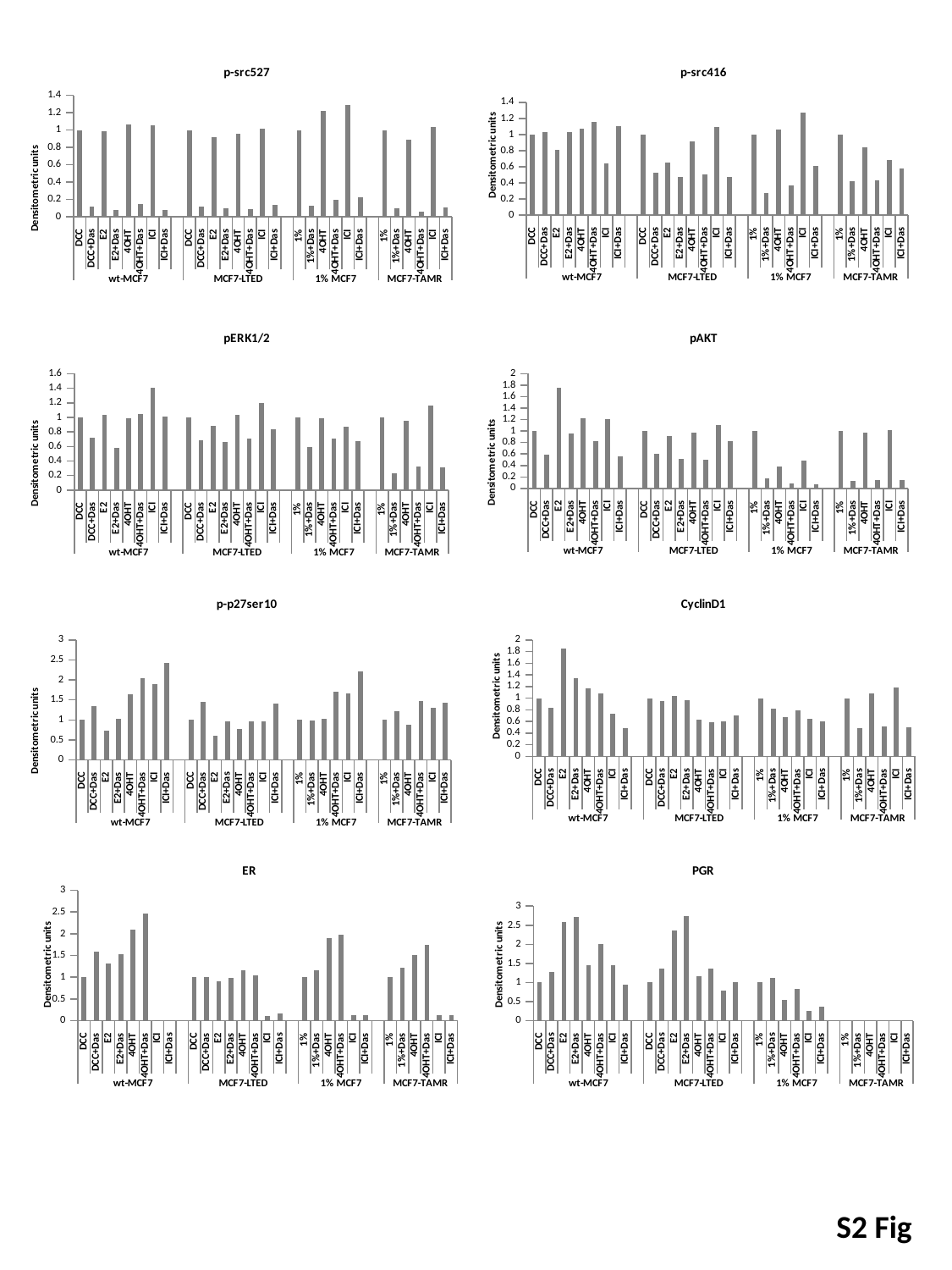

### Chart:
| Category | p-src527 |
|---|---|
| DCC | 1.0 |
| DCC+Das | 0.113729235045812 |
| E2 | 0.9824650977762 |
| E2+Das | 0.080451727916125 |
| 4OHT | 1.060136254055643 |
| 4OHT+Das | 0.147987931454625 |
| ICI | 1.056513955610471 |
| ICI+Das | 0.0834094399117263 |
| | 0.0 |
| DCC | 1.0 |
| DCC+Das | 0.113890379835452 |
| E2 | 0.913279440937964 |
| E2+Das | 0.101477444883871 |
| 4OHT | 0.956694406401639 |
| 4OHT+Das | 0.0887419505563425 |
| ICI | 1.01029876778114 |
| ICI+Das | 0.134292150810178 |
| | 0.0 |
| 1% | 1.0 |
| 1%+Das | 0.125719417523726 |
| 4OHT | 1.221690819011608 |
| 4OHT+Das | 0.191999999596951 |
| ICI | 1.28496277775431 |
| ICI+Das | 0.228226699158322 |
| | 0.0 |
| 1% | 1.0 |
| 1%+Das | 0.0944154365103635 |
| 4OHT | 0.888511549632913 |
| 4OHT+Das | 0.0625306004426141 |
| ICI | 1.032015436336156 |
| ICI+Das | 0.105739948429413 |
### Chart:
| Category | p-src416 |
|---|---|
| DCC | 1.0 |
| DCC+Das | 1.031038696364617 |
| E2 | 0.807216274278276 |
| E2+Das | 1.031071921296063 |
| 4OHT | 1.072335873627654 |
| 4OHT+Das | 1.152362450692032 |
| ICI | 0.647141368059903 |
| ICI+Das | 1.10154386435777 |
| | 0.0 |
| DCC | 1.0 |
| DCC+Das | 0.524801379928044 |
| E2 | 0.64872057246692 |
| E2+Das | 0.475712479006393 |
| 4OHT | 0.914452862989843 |
| 4OHT+Das | 0.510571844950421 |
| ICI | 1.091056058095947 |
| ICI+Das | 0.468946466051015 |
| | 0.0 |
| 1% | 1.0 |
| 1%+Das | 0.276725053959688 |
| 4OHT | 1.058396766267005 |
| 4OHT+Das | 0.373960351007052 |
| ICI | 1.269046104579736 |
| ICI+Das | 0.614211012748669 |
| | 0.0 |
| 1% | 1.0 |
| 1%+Das | 0.421703796087414 |
| 4OHT | 0.837421260732012 |
| 4OHT+Das | 0.427106693021135 |
| ICI | 0.680571587840221 |
| ICI+Das | 0.576558562578616 |
### Chart:
| Category | pERK1/2 |
|---|---|
| DCC | 1.0 |
| DCC+Das | 0.724846081778909 |
| E2 | 1.039521164939927 |
| E2+Das | 0.585962316461821 |
| 4OHT | 0.994566445022065 |
| 4OHT+Das | 1.048869241884305 |
| ICI | 1.405351974281131 |
| ICI+Das | 1.00794039423153 |
| | 0.0 |
| DCC | 1.0 |
| DCC+Das | 0.684093839096644 |
| E2 | 0.88577470239088 |
| E2+Das | 0.658156444592807 |
| 4OHT | 1.041299407904853 |
| 4OHT+Das | 0.709205347326261 |
| ICI | 1.196243505142684 |
| ICI+Das | 0.841847137930491 |
| | 0.0 |
| 1% | 1.0 |
| 1%+Das | 0.596864586030708 |
| 4OHT | 0.98537109736997 |
| 4OHT+Das | 0.713264813784905 |
| ICI | 0.869199713666559 |
| ICI+Das | 0.672190319613961 |
| | 0.0 |
| 1% | 1.0 |
| 1%+Das | 0.236913143065989 |
| 4OHT | 0.957296103372798 |
| 4OHT+Das | 0.323175622462141 |
| ICI | 1.168509320251043 |
| ICI+Das | 0.311998998654859 |
### Chart:
| Category | pAKT |
|---|---|
| DCC | 1.0 |
| DCC+Das | 0.584899017707593 |
| E2 | 1.750476237234482 |
| E2+Das | 0.956512224621084 |
| 4OHT | 1.219030752962945 |
| 4OHT+Das | 0.823909989466533 |
| ICI | 1.214127031577555 |
| ICI+Das | 0.561952146994519 |
| | 0.0 |
| DCC | 1.0 |
| DCC+Das | 0.596965022025561 |
| E2 | 0.910994627898936 |
| E2+Das | 0.517319001768344 |
| 4OHT | 0.967205996793291 |
| 4OHT+Das | 0.504753408548655 |
| ICI | 1.106657814292974 |
| ICI+Das | 0.824121633976825 |
| | 0.0 |
| 1% | 1.0 |
| 1%+Das | 0.178570636059021 |
| 4OHT | 0.375384475675662 |
| 4OHT+Das | 0.0814175099190161 |
| ICI | 0.478561780414138 |
| ICI+Das | 0.072162620071785 |
| | 0.0 |
| 1% | 1.0 |
| 1%+Das | 0.137134885266423 |
| 4OHT | 0.968922812269888 |
| 4OHT+Das | 0.144401895956016 |
| ICI | 1.013286312602537 |
| ICI+Das | 0.149956004743365 |
### Chart:
| Category | p-p27ser10 |
|---|---|
| DCC | 1.0 |
| DCC+Das | 1.349613232583923 |
| E2 | 0.73899251298108 |
| E2+Das | 1.018579685560592 |
| 4OHT | 1.636849506679748 |
| 4OHT+Das | 2.042462300987944 |
| ICI | 1.901933922916319 |
| ICI+Das | 2.4166006279265 |
| | 0.0 |
| DCC | 1.0 |
| DCC+Das | 1.445160621396871 |
| E2 | 0.610520602757456 |
| E2+Das | 0.967986739496576 |
| 4OHT | 0.76289999828971 |
| 4OHT+Das | 0.960354048561696 |
| ICI | 0.970664291028977 |
| ICI+Das | 1.404762421461485 |
| | 0.0 |
| 1% | 1.0 |
| 1%+Das | 0.980784690644917 |
| 4OHT | 1.037185021720568 |
| 4OHT+Das | 1.705133365133825 |
| ICI | 1.668792005828003 |
| ICI+Das | 2.216075017571649 |
| | 0.0 |
| 1% | 1.0 |
| 1%+Das | 1.207577659992309 |
| 4OHT | 0.869469311484861 |
| 4OHT+Das | 1.47575461355032 |
| ICI | 1.307524858597896 |
| ICI+Das | 1.428042749557647 |
### Chart:
| Category | CyclinD1 |
|---|---|
| DCC | 1.0 |
| DCC+Das | 0.837094823034671 |
| E2 | 1.845918122379924 |
| E2+Das | 1.350131567804689 |
| 4OHT | 1.174955662923997 |
| 4OHT+Das | 1.075263845590619 |
| ICI | 0.734392343840526 |
| ICI+Das | 0.490955104751499 |
| | 0.0 |
| DCC | 1.0 |
| DCC+Das | 0.944977407270957 |
| E2 | 1.042043260735848 |
| E2+Das | 0.963609213456664 |
| 4OHT | 0.634857025253845 |
| 4OHT+Das | 0.593404367194479 |
| ICI | 0.604094814096352 |
| ICI+Das | 0.708621566279603 |
| | 0.0 |
| 1% | 1.0 |
| 1%+Das | 0.823717552729363 |
| 4OHT | 0.669046006472528 |
| 4OHT+Das | 0.791133895397601 |
| ICI | 0.644231220396108 |
| ICI+Das | 0.595373217314598 |
| | 0.0 |
| 1% | 1.0 |
| 1%+Das | 0.478569498147079 |
| 4OHT | 1.081456842752534 |
| 4OHT+Das | 0.511567920062206 |
| ICI | 1.177954668866775 |
| ICI+Das | 0.498743268420362 |
### Chart:
| Category | ER |
|---|---|
| DCC | 1.0 |
| DCC+Das | 1.592739372044074 |
| E2 | 1.315946876109725 |
| E2+Das | 1.526044059143451 |
| 4OHT | 2.091583874649587 |
| 4OHT+Das | 2.473198099641146 |
| ICI | 0.00671560881661418 |
| ICI+Das | 0.0 |
| | 0.0 |
| DCC | 1.0 |
| DCC+Das | 1.004641455260712 |
| E2 | 0.903812000066279 |
| E2+Das | 0.994226202577765 |
| 4OHT | 1.153543364554654 |
| 4OHT+Das | 1.034562341479963 |
| ICI | 0.112220241701952 |
| ICI+Das | 0.166441661611735 |
| | 0.0 |
| 1% | 1.0 |
| 1%+Das | 1.157238883832335 |
| 4OHT | 1.896856478424373 |
| 4OHT+Das | 1.979067267134591 |
| ICI | 0.122244869148719 |
| ICI+Das | 0.133459547672597 |
| | 0.0 |
| 1% | 1.0 |
| 1%+Das | 1.211029219067985 |
| 4OHT | 1.508113583902565 |
| 4OHT+Das | 1.752595722026294 |
| ICI | 0.130565072692593 |
| ICI+Das | 0.119938663692263 |
### Chart:
| Category | PGR |
|---|---|
| DCC | 1.0 |
| DCC+Das | 1.267483017083687 |
| E2 | 2.57933420713514 |
| E2+Das | 2.709518494405607 |
| 4OHT | 1.44748313430351 |
| 4OHT+Das | 1.998620160976041 |
| ICI | 1.44660902676465 |
| ICI+Das | 0.945223376037972 |
| | 0.0 |
| DCC | 1.0 |
| DCC+Das | 1.371020474212283 |
| E2 | 2.369321336588943 |
| E2+Das | 2.73589483112709 |
| 4OHT | 1.174367039725111 |
| 4OHT+Das | 1.374722004443413 |
| ICI | 0.793594419885945 |
| ICI+Das | 1.009735373686022 |
| | 0.0 |
| 1% | 1.0 |
| 1%+Das | 1.126746238462571 |
| 4OHT | 0.552033940765455 |
| 4OHT+Das | 0.823208168983193 |
| ICI | 0.263382199055458 |
| ICI+Das | 0.373024909575783 |
| | 0.0 |
| 1% | 0.0 |
| 1%+Das | 0.0 |
| 4OHT | 0.0 |
| 4OHT+Das | 0.0 |
| ICI | 0.0 |
| ICI+Das | 0.0 |S2 Fig
