# Supplementary material for: Src Is a Potential Therapeutic Target in Endocrine-Resistant Breast Cancer Exhibiting Low Estrogen Receptor-Mediated Transactivation
Source: PLoS One. 2016 Jun 16;11(6):e0157397. doi: 10.1371/journal.pone.0157397 (PMC4911087; doi:10.1371/journal.pone.0157397)
Supplement: S3 Fig — Bars represent ± SEM. *p<0.05, **p<0.01, ***p<0.001. (PPTX) [file pone.0157397.s003.pptx]

## Slide 1
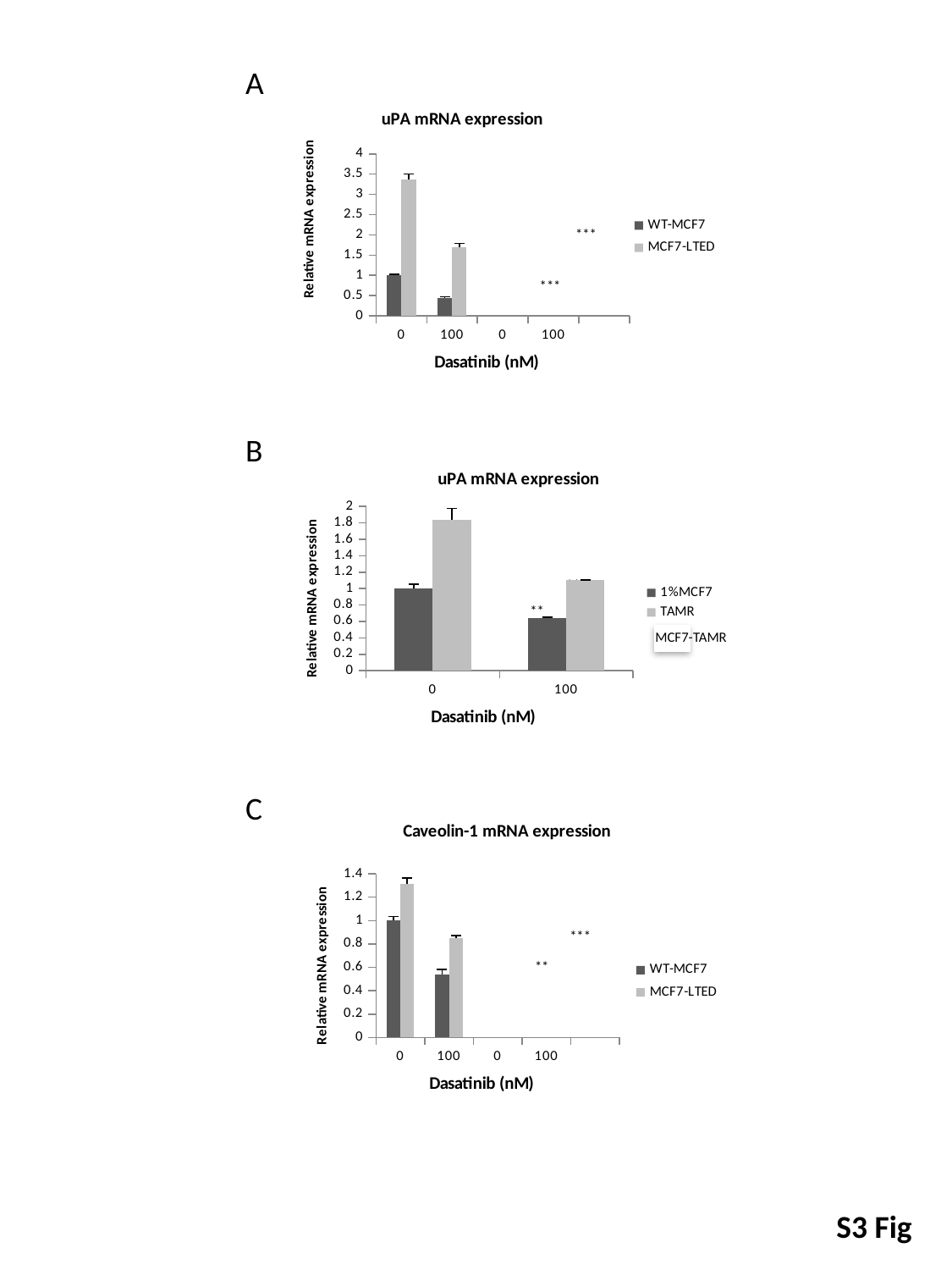

A
[unsupported chart]
***
***
B
### Chart: uPA mRNA expression
| Category | 1%MCF7 | TAMR |
|---|---|---|
| 0.0 | 1.0 | 1.837448429975915 |
| 100.0 | 0.642991056239049 | 1.101150551902677 |MCF7-TAMR
**
**
C
[unsupported chart]
***
**
S3 Fig
