# Supplementary material for: Src Is a Potential Therapeutic Target in Endocrine-Resistant Breast Cancer Exhibiting Low Estrogen Receptor-Mediated Transactivation
Source: PLoS One. 2016 Jun 16;11(6):e0157397. doi: 10.1371/journal.pone.0157397 (PMC4911087; doi:10.1371/journal.pone.0157397)
Supplement: S4 Fig — Baseline expression of proteins in MCF7-LTED and MCF7-TAMR compared to their wt counterparts Figures below each panel represent semi-quantitave changes in protein expression relative to actin (A). ER/ERE transactivation in wt-MCF7, MCF7-LTED, 1%MCF7 and MCF7-TAMR cells (B). Bars represent ± SEM. *p<0.05, **p<0.01, ***p<0.001. (PPTX) [file pone.0157397.s004.pptx]

## Slide 1
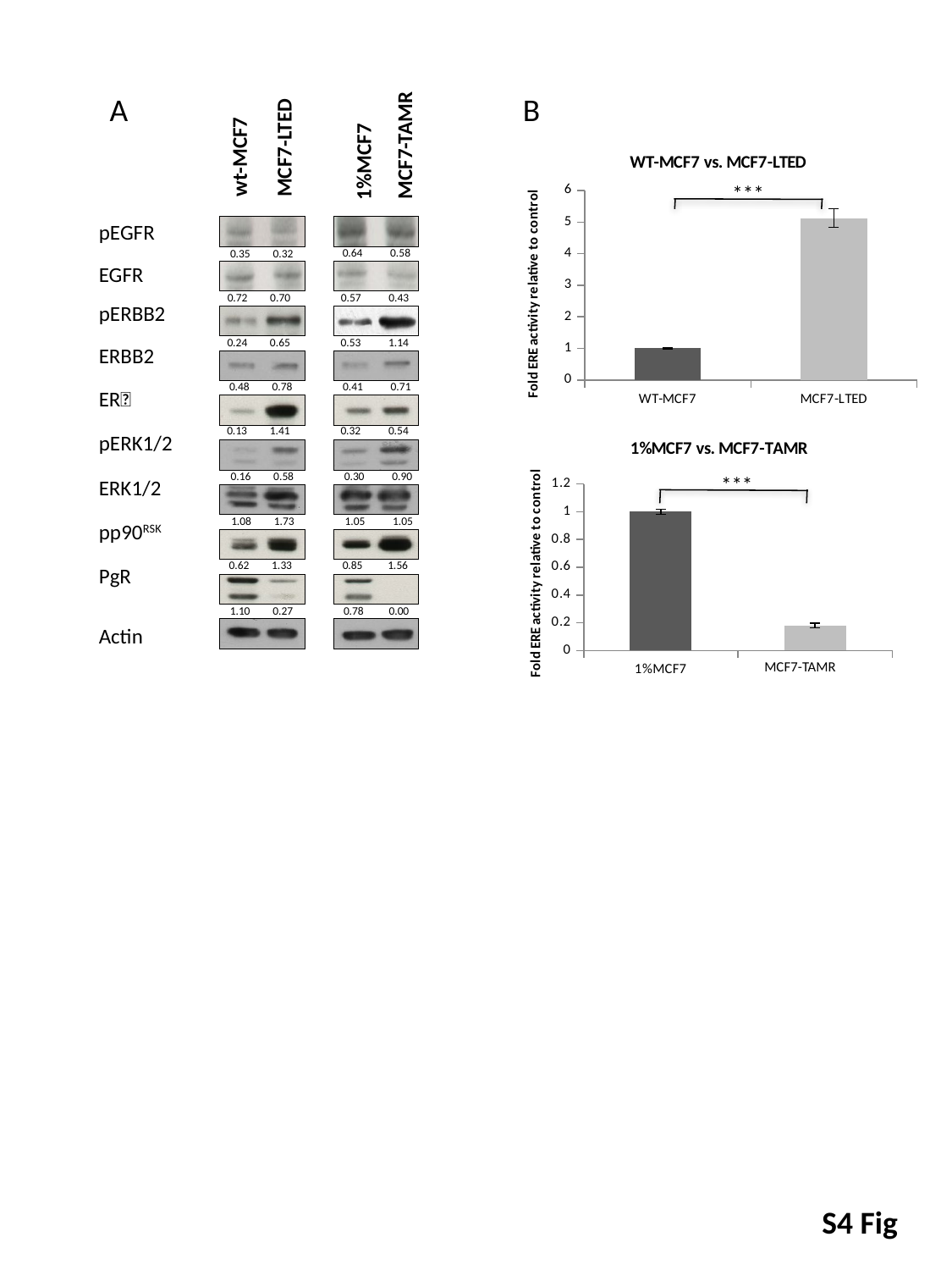

A
B
MCF7-LTED
wt-MCF7
MCF7-TAMR
1%MCF7
### Chart: WT-MCF7 vs. MCF7-LTED
| Category | |
|---|---|
| WT-MCF7 | 1.0 |
| MCF7-LTED | 5.127123833512266 |***
pEGFR
0.64 0.58
0.35 0.32
EGFR
0.72 0.70
0.57 0.43
pERBB2
0.24 0.65
0.53 1.14
ERBB2
0.48 0.78
0.41 0.71
ERα
0.13 1.41
0.32 0.54
### Chart: 1%MCF7 vs. MCF7-TAMR
| Category | |
|---|---|
| 1%MCF7 | 1.0 |
| TAMR | 0.180965130998744 |MCF7-TAMR
pERK1/2
0.16 0.58
0.30 0.90
***
ERK1/2
1.08 1.73
1.05 1.05
pp90RSK
0.62 1.33
0.85 1.56
PgR
1.10 0.27
0.78 0.00
Actin
S4 Fig
