# Supplementary figures and images for: Src Is a Potential Therapeutic Target in Endocrine-Resistant Breast Cancer Exhibiting Low Estrogen Receptor-Mediated Transactivation
Source: PLoS One. 2016 Jun 16;11(6):e0157397. doi: 10.1371/journal.pone.0157397 (PMC4911087; doi:10.1371/journal.pone.0157397)

## Slide 1
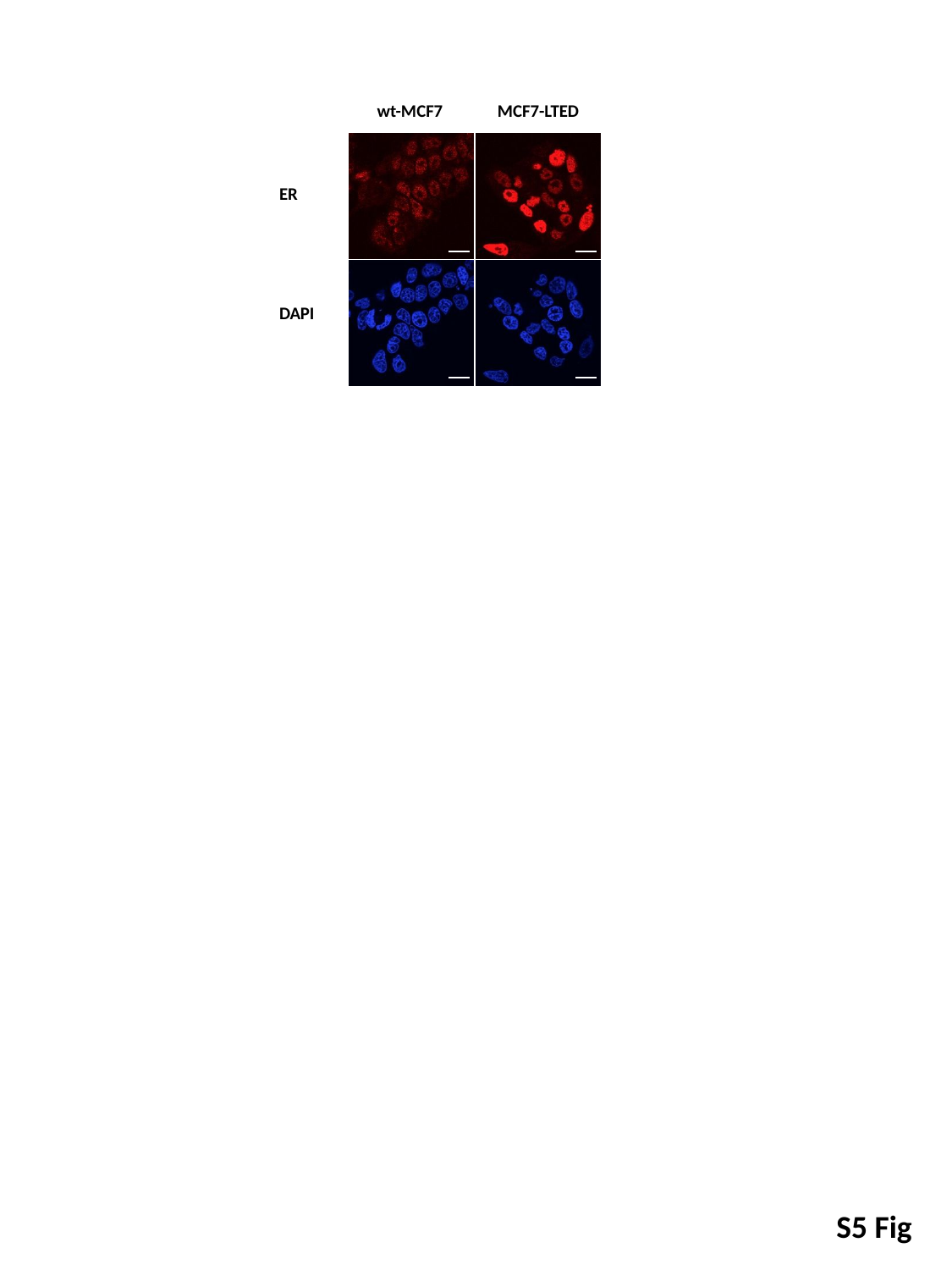

wt-MCF7
MCF7-LTED
ER
DAPI
S5 Fig

Supplement: S5 Fig — (PPTX) [file pone.0157397.s005.pptx]
